# Brand Suicide? Memory and Liking of Negative Brand Names

**DOI:** 10.1371/journal.pone.0151628

**Published:** 2016-03-29

**Authors:** Duncan Guest, Zachary Estes, Michael Gibbert, David Mazursky

**Affiliations:** 1 Psychology Division, Nottingham Trent University, Nottingham, United Kingdom; 2 Department of Marketing, Bocconi University, Milan, Italy; 3 Institute of Marketing and Communication, University of Lugano, Lugano, Switzerland; 4 The Jerusalem School of Business Administration, The Hebrew University Of Jerusalem, Jerusalem, Israel; 5 The Centre for Academic Studies, Or Yehuda, Israel; The University of Nottingham, UNITED KINGDOM

## Abstract

Negative brand names are surprisingly common in the marketplace (e.g., *Poison* perfume; *Hell* pizza, and *Monster* energy drink), yet their effects on consumer behavior are currently unknown. Three studies investigated the effects of negative brand name valence on brand name memory and liking of a branded product. Study 1 demonstrates that relative to non-negative brand names, negative brand names and their associated logos are better recognised. Studies 2 and 3 demonstrate that negative valence of a brand name tends to have a detrimental influence on product evaluation with evaluations worsening as negative valence increases. However, evaluation is also dependent on brand name arousal, with high arousal brand names resulting in more positive evaluations, such that moderately negative brand names are equally as attractive as some non-negative brand names. Study 3 shows evidence for affective habituation, whereby the effects of negative valence reduce with repeated exposures to some classes of negative brand name.

## Introduction

You are choosing a wine for dinner in a restaurant. The waiter hands you the wine list and among the usual Old and New World wines, such as a Chablis by *Louis Latour* and *Stag’s Leap* cabernet sauvignon, there are some curiously negative brand names. One wine is called *Frog’s Piss* (a French red table wine), and another one *Fat Bastard* (a French Chardonnay blend). When looking for a Chardonnay would you go for the *Fat Bastard* or rather stick to the *Louis Latour*? In this article, we examine when brand names that are negative, such as *Fat Bastard* might appeal.

Negatively named new brands as well as extensions of existing brands inconspicuously seem to have sprung up in myriad product categories (e.g., Coca Cola’s *Monster* energy drink, *Criminal* clothing, and the *Heart Attack Grill* in Las Vegas, Nevada to name but a few). This phenomenon has even drawn recent attention in the media, with an article in the New York Times (2011) [1] on notoriously negative wine labels sporting *Sassy Bitch*, *Ball Buster*, and *BigAss Red*. Intuitively, using negative words as brand names seems outright bizarre. Cognitive research confirms this, showing that negative words generate negative feelings, signal threats [2,3] and therefore lead to avoidance responses [4,5]. Clearly, none of these effects are desirable characteristics for a brand name.

Despite this, brand names with negative valence have actually been around for decades, a well-known example being Dior’s *Poison* perfume (introduced in 1985). Evidently something must make them appealing–at least to some people. A case in point is *Fat Bastard*. *Businessweek* recently called *Fat Bastard* a marketing phenomenon, selling over 400,000 cases in the year following its introduction, in the US alone. In 2002 Dior launched another fragrance with a negative brand name, *Addict*, indicating that there is something about such names that marketers believe appeal. In view of the many examples of negative brand names, both established and more recent, alongside the existence of a literature on brand name effects [6–8] it seems surprising that practically nothing is known about why they might appeal to customers. The research presented here opens up this area of research. In this article we consider the importance of the characteristics of the actual brand name (i.e., its negative valence) and assess the role this could play in the success of negative brand names. In so doing, we agree with Scott et al [9] who note that emotion research in psycholinguistics has focused on how we process words, but that research on the role of emotion in persuasion has focused on the effects of emotional language in general, rather than the effects of emotional content of a given word. It is therefore important to understand the effects of these individual emotional words, particularly in the case of brand names, given their prominence in marketing.

### Negative Brand Names Activate Automatic Vigilance

The issue of how we process valence has received much attention in psychology because negative/positive stimuli potentially signal a threat/reward. To ensure our survival, it is important that we react to potential threats. According to this *automatic vigilance*, negative information may have undesirable consequences for the perceivers’ wellbeing and so is automatically, preferentially, and more efficiently processed [3,10–14]. For instance, negative words capture and hold attention [12,15,16] and induce larger neural responses than positive words [17,18].

This automatic vigilance also influences memory. Relative to non-negative words, words with negative valence are better recognised and recalled [19,20]. Memory is improved not just for negative words themselves, but also for characteristics of these words, such as the colour font [20]. Moreover, besides capturing attention, it also takes longer to disengage attention from negative words [10]. Based on this research it seems possible then that using a negative word as a brand name may have distinct advantages in terms of attentional capture and memorability. In the first of the studies in this article we therefore examine whether real brand names that are negative in valence are more likely to be remembered than brand names that are non-negative in valence. Importantly, our experiments focus on the influence of negative brand names relative to non-negative brand names, that is, brand names with an average level of valence that cannot easily be labelled positive or negative in valence. Comparing the effects of negative and non-negative brand names, we believe, is the most straightforward in terms of understanding the effects of the negative valence of brand names. Moreover, there are large numbers of non-negative brand names in existence, making this a useful comparison. However, it is worth noting that a large amount of research on the effects of positive valence on word processing also exists. Positive valence has been shown to have a variety of effects, for example leading to faster lexical decision than neutral words [15,21], and neurologically there has been some evidence that positive words receive a processing advantage and they appear to activate different neurological structures than negative words [22]. Whilst the role of positive valence of brand names is interesting, a trend towards the use of negative brand names in recent years makes this phenomenon particularly interesting. As such, we focus on the comparison between negative and non-negative brand names.

As well as looking at word recognition, the first study also examines memory for characteristics of brand names, specifically the colour in which the brand name is graphically represented. In addition, we examine memory for a logo associated with a brand name. Previous research on word valence effects on memory has focused on memory for words or characteristics of the words [23]. Thus it is possible that negative valence leads to selective processing of the word only. Alternatively word valence effects may not be limited to words themselves but could generalise to other visual attributes presented in close proximity to the word. Indeed Mackay and colleagues [24–26] suggest that emotional stimuli enhance memory for contextual details, not simply characteristics of the words themselves. Their binding hypothesis suggests that emotional stimuli activate binding mechanisms that bind the source of the emotion with features of the surrounding context. This results in better memory for characteristics of the word such as colour [25] as well as other contextual details such as memory for the location at which the word was presented [26]. Memory for logos that are associated by their proximity with negative brand names should therefore be better recognised than logos associated with non-negative brand names.

Vigilance for negative information may also have effects on product perception and evaluation. Indeed, recent research has shown that simply altering the valence (positive or negative) of words within a product message influences product perceptions [9]. Interestingly, the effect differed depending on the context of the message. Compared to positive words, messages containing negative words produced more positive ratings when they were contained within a promotion-focused message, but worse ratings when within a message focusing on prevention (minimising losses). In contrast, brand names are often presented in isolation, and tend to be the first point of contact with a brand. It is therefore important to consider the effects of negative brand name valence of product perception.

What might the effect of negative brand names on product perception be? By their very nature, negative words will be associated with negative concepts and undesirable consequences that will become activated when processing a negative brand name [27]. Additionally negative words are potentially threatening and can trigger an avoidance response [4,5,13]. Given the detrimental impact negative affect has upon a range of consumer behaviours [28–34] utilizing a negative brand name might be expected to worsen product evaluations. Valence is a continuous dimension however (e.g., moderate to extremely negative) and so this might mean that there are differing effects of negative valence as the negative valence of the brand name increases in extremity. In two of the largest studies to date on automatic vigilance, extremely negative words elicited slower responses [15] and were better remembered [21] than moderately negative words. Thus, extremely negative brand names may produce a much greater affective response and create greater avoidance than moderately negative words. In contrast, brand names which are only moderately negative may produce a milder affective response, and so their influence on product evaluation will be reduced compared to extremely negative brand names.

In three studies, we examine the effects of negative valence. Study 1 examined whether brand names, their perceptual characteristics (colour) and their associated logos are better remembered when brand names have negative valence compared to non-negative valence. Studies 2 and 3 examine the effect of negative brand names on product perception.

## Study 1

### Method

#### Participants

Participants were 84 undergraduate students at a typical European university who participated voluntarily as part of a class exercise. All participants were over 18 and the majority under the age of 25, studying in English for a postgraduate degree. The experiment materials and instructions were all in English.

#### Materials and Stimuli

In a pretest we asked students to list actual brand names they had heard of that had a negative valence. From this list we generated a set of 20 negative brand names that were independently verified as existing in the marketplace (see Table 1). Each of these brand names contained a negative word, as measured by the Affective Norms for English Words (ANEW, [35]). The ANEW contains a list of words that have been extensively rated for their valence (from 1 (negative) to 9 (positive)) and arousal (from 1 (not arousing) to 9 (highly arousing)) and is widely used in psycholinguistic research. Where there was not an exact match of the negative word in the ANEW we used the nearest equivalent (e.g., *fight* rather than *fighter*). Non-negative brand names were also selected from the ANEW. Mean valence was 2.64 for the negative words and 4.60 for the non-negative words, and this difference was statistically significant, *t*(38) = 12.06, *p* < .001 (the ANEW valence ratings for each stimulus are shown in Table 1). Because word length and frequency affect word recognition, we selected non-negative words that were matched with the negative words for both log word frequency (*t*(38) = 1.78, *p* = .38), and length (*t*(38) = 1.78, *p* = .08) (see Table 1). Word frequency, which is the number of occurrences of a given word in a massive text corpus, is an objective measure of familiarity. In fact, word recognition is better predicted by objective frequency than by subjective familiarity [36]. It is typical in psycholinguistics to control for log frequency, rather than frequency per se, due to the extreme skew in the frequency distribution. Thus, the negative and non-negative words differed significantly in valence but were equal in length and frequency.

**Table 1:** Non-negative and negative brand names from study 1.

Real brand names(product)	Experimental brand names							
	Negative				Non-Negative			
		Valence	Arousal	Frequency (Log)		Valence	Arousal	Frequency (Log)
*Criminal (clothing)*	criminal	2.93	4.79	9.88	register	4.95	4.00	9.94
*Fat Face (clothing)*	fat	2.28	4.81	10.29	radiator	4.67	4.02	6.96
*Loser (clothing)*	loser	2.25	4.95	8.34	insect	4.07	4.07	7.84
*Red or Dead (clothing)*	dead	1.94	5.73	11.2	contents	4.89	4.32	10.02
*No Fear (clothing)*	fear	2.76	6.96	10.45	storm	4.95	5.71	9.90
*Murder (clothing)*	murderer	1.53	7.47	7.60	chaos	4.17	6.67	9.87
*Killer (clothing)*	killer	1.89	7.86	9.52	anxious	4.81	6.92	7.91
*Firetrap clothing*	fire	3.22	7.17	11.05	volcano	4.84	6.33	8.02
*Tornado (clothing and cosmetics)*	tornado	2.55	6.83	7.42	razor	4.81	5.36	8.09
*Poison (perfume)*	poison	1.98	6.05	8.54	stomach	4.82	3.93	8.95
*Burn (energy drink)*	burn	2.73	6.22	9.43	army	4.72	5.03	10.44
*Shark (energy drink)*	shark	3.65	7.16	8.09	pistol	4.2	6.15	8.45
*Fat bastard (wine)*	bastard	3.36	6.07	8.45	kerosene	4.8	4.34	6.36
*Hell pizza (restauraunt)*	hell	2.24	5.38	11.03	dark	4.71	4.28	11.17
*Agusta Brutale (motorbike)*	brutal	2.80	6.60	8.08	cold	4.02	5.19	10.46
*Ducatti Street fighter (motorbike)*	fight	3.76	7.15	10.6	dentist	4.02	5.73	7.30
*Cyclone(microsystems)*	cyclone	3.60	6.36	7.97	lesbian	4.67	5.12	8.94
*Demon (internet provider)*	demon	2.11	6.76	9.10	hide	4.32	5.28	9.55
*Danger Inc*. *(internet)*	danger	2.95	7.32	9.45	obsession	4.52	6.41	7.77
*Black Devil (cigarettes)*	devil	2.21	6.07	9.02	payment	4.95	4.95	10.33
**Mean**		**2.64**	**6.39**	**9.27**		**4.6**	**5.19**	**8.91**

Although the words in the negative and non-negative list were balanced for word frequency affect is thought to be determined jointly by valence and arousal and arousal effects on word processing are well established [15,22]. Mean arousal ratings for negative words (6.39) were significantly greater than non-negative words (5.19), (*t*(38) = 4.05, *p* < .001). The unique contributions of arousal and valence are therefore assessed in the analysis.

#### Design and Procedure

**Fig 1:**
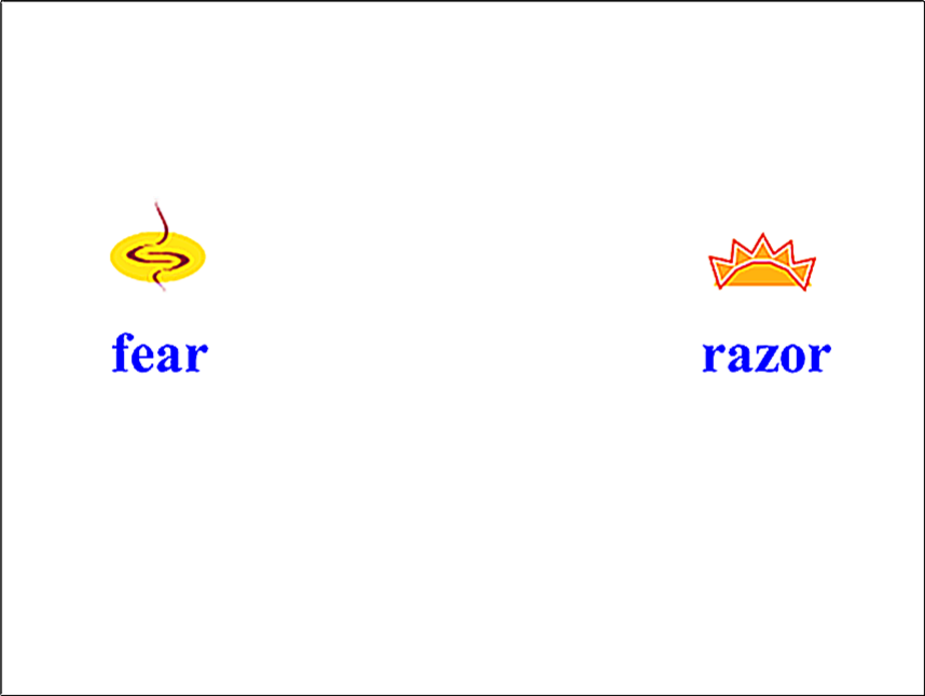
An example of a slide shown in study 1.

During the study stimuli were shown on a PowerPoint presentation in which each slide contained two words (one non-negative and one negative), one on the left and one on the right (assignation of brand name position was random), with a logo presented above each word (see Fig 1A). Words were either red or blue in colour. Participants were not told that the words were brand names to ensure that brand name familiarity did not bias attention. 20 of these slides were shown for 5 seconds each. Participants were told they would be shown a series of slides (each shown for 5s) that would contain some words and pictures. They were told to pay attention to these and that afterward some questions would be asked about what they had seen. They were therefore not explicitly informed that there would be a memory test, and thus learning should have been incidental (as it is in everyday life). After stimulus presentation, respondents were shown 60 logos for three seconds each. 20 had been presented with negative words, 20 had been presented with non-negative words and 20 were new logos that had not been shown previously. For each logo, respondents indicated whether they had seen the logo in the previous presentation. After the logo recognition test, respondents then saw a list of 40 words presented individually in black font for 3 seconds each. 20 of these were seen in the earlier presentation (half negative, half non-negative), and the other 20 were new words not seen in the presentation (half with non-negative valence, half with negative valence). Respondents again indicated whether they had seen the word or not in the previous presentation. In the final task 20 words were presented individually for 3 seconds in either red or blue ink. All the words had been shown in the initial presentation (half non-negative and half negative) and half of the items were shown in the same colour as they had been in the initial presentation. Respondents indicated whether the words were in the same colour as they had been in the earlier presentation.

#### Ethics statement

Participants in this and the following studies were sampled via an opportunity sample. The researcher asked members of staff in the department for permission to come to one of their lecture classes and ask their students whether they would take part in a short study. The researcher then attended a randomly selected class from those available Each study reported here sampled participants from different lecture classes and each study sampled students from only one lecture class. Importantly, the researcher did not teach the students, the class was unrelated to the study and there was no course incentive to complete the research. At the beginning of a class students were asked if they would like to take part in a short study. Those willing to take part gave their oral consent and were issued with the necessary response sheet. Participants did not have to complete the task and were instructed that they could withdraw at any time. Data was anonymised by participants noting down a unique identifier that only they were aware of. This also served as recording of their oral consent. Oral consent was assumed sufficient given the non-medical, non-intrusive and zero risk nature of task (recognising words and logos in Study 1 and evaluating branded products in Studies 2–3). At the time of data collection the institutions at which data collection occurred did not have an ethics review board and so the study could not be reviewed or approved by one. As there was no ethical review board, the procedure and use of oral consent was not evaluated.

### Results and Discussion

Relative to non-negative brand names such as *Storm*, negative brand names such as *Poison* elicited superior recognition of: the brand name itself both in terms of percentage correct (*F*(83) = 19.25, *η*^*2*^.19, *p* < .001) and *d’* (*F*(83) = 7.53, *η*^*2*^.083, *p* = .007) as well as its colour (*F*(83) = 5.99, *η*^*2*^.067, *p* = .017) and its associated logo (*F*(83) = 7.06, *η*^*2*^.078, *p* = .009) (see Table 2).

**Table 2:** Recognition data (mean percentage correct and mean *d’* with standard deviations in brackets) for non-negative and negative brand names from study 1.

	Negative brand names	Non-negative brand names
Word recognition (% correct)	74.2% (.17)	65.0% (.19)
*d’*	1.97 (.93)	1.65 (.95)
Word colour recognition (% correct)	54.6% (.20)	49.0% (.22)
Logo recognition (% correct)	58.0% (.16)	53.6% (.15)

However, stimuli differed in terms of arousal as well as valence, with the negative brand names being more arousing. Notably, in addition to valence, affect is also determined by arousal. Marketing research has shown that, independently of valence, stimuli that are highly arousing (e.g., the shopping environment, ambient scents, the service setting) can lead to positive effects, such as spending level, satisfaction and repurchase intention [37,38]. We therefore performed a post-hoc analysis to determine whether the observed effects were driven by arousal. In the first analyses the negative and non-negative sets were split into two based on arousal levels, resulting in a high and average arousal group of items within each set. The negative average arousal set were then compared with the non-negative high arousal set. Arousal, log word frequency or word length did not differ between these sets (all *t*<1.4) but valence did (*t*(18) = 8.86, *p* < .001). On this subset of stimuli negative brand names elicited superior recognition of word colour (*F*(1, 83) = 15.23, *η*^*2*^.16, *p* < .001) and of logos associated with negative words (*F*(1, 83) = 50.74, *η*^*2*^.38, *p* < .001). However there was no significant effect of word valence on word recognition in terms of percentage correct (*F*(1, 83) = 1.32, *η*^*2*^ = .016, *p* < .26) or *d’* (*F*(1, 83) = .85, *η*^*2*^.01, *p* = .36).

In addition, an item analysis was conducted by a multiple regression examining the extent to which arousal, valence, log frequency and word length predicted recognition memory for the previously presented logos. For proportion correct logo recognition the regression model accounted for a significant amount of variance (*F*(4, 35) = 3.9, *p* < .010) and accounted for 23% of the variance (adjusted *r*^*2*^ = .23) in logo recognition. Significant predictors were valence (beta = -.08, *p* = .004) and arousal (beta = -.09, *p* = .002). Thus, logo recognition increased as arousal decreased and valence became more negative. For proportion correct word colour recognition the regression model was not significant (*F*(4, 15) = 1.58, *p* = .23), perhaps unsurprising given the small number of items in the analysis (twenty). However, within the analysis valence was the only the only significant predictor (beta = -.67, *p* = .028) such that better colour recognition was associated with more negative words. For proportion correct word recognition (for both old items and the new items) the regression model was significant (*F*(4, 35) = 4.65, *p* < .004) but none of the predictors had a significant effect (the fit of the model was driven by the constant). Importantly, caution should be taken when interpreting these regression analyses as they were considerably more underpowered than the participant analysis. Nevertheless, they highlight that when controlling for levels of arousal, word length and word frequency, independent effects of valence on logo recognition and colour recognition were observed, such that decreasing valence led to greater recognition.

Overall, then, it is clear then that one benefit of using a brand name with negative valence is that the negative valence may improve recognition. Here we found that, when it is put in direct competition with a non-negative word by presenting it within the same display, recognition is better for the negative word, characteristics of it such as its colour and its associated logo. Although some of the effects in study 1 may have been driven by the negative items being more arousing, an independent effect of negative valence was observed on logo recognition and word colour recognition, indicating at the very least that negative valence may make a brand name more recognisable when its colour is consistent and improve memory for its associated logo. An important feature of study 1 is that negative and non-negative stimuli were put in direct competition. As such the results may have been driven by attentional capture of the negative words relative to the non-negative words. It is possible then that presenting stimuli in isolation would yield different results. However effects of negative valence on word recognition have been observed when presenting stimuli sequentially (21) and in real life brand names are often presented in situations where they compete for attention with non-negative brand names.

Recent research of the effects of valence and arousal on memory suggest that they might have different effects on conscious recollection (remembering features relating to a prior experience of the stimulus) or non-recollection (a sense of familiarity without conscious recollection). Thus valence may improve memory not because it increases conscious recollection, but because it increases familiarity [39,40]. Specifically, negative words are remembered better than neutral words but this effect is driven by non-recollection [39]. Although our methodology does not allow for discrimination between recollection and non-recollection, it is quite possible that the greater recognition for negative brand names in study 1 was driven by non-recollection. Indeed, that could partially explain why the effect of valence independent of arousal was observed for colour recognition and logo recognition, as these would not have been verbally encoded during stimulus presentation and thus responses would be more based upon familiarity. In contrast, the words would have been verbally encoded and so participants may have been trying to base their decisions more on conscious recollection.

Overall then, using a negative brand name can have a positive effect on memory, and thus potential positive effects on brand awareness. However this benefit may come at a cost. Negative brand names may well have a detrimental impact on evaluations of a branded product due to their associations with other negative things that may be automatically elicited upon processing the name and because negative valence can produce an avoidance response. The central aim of study 2 was therefore to examine the effect of brand name valence on evaluation of branded products. As valence is continuous not categorical [35] the extent of the negative valence of the brand name was manipulated. Even more importantly, because affect is determined by both valence and arousal and we suspected that they might interact in brand name evaluation, we also manipulated brand name affect independently of valence.

Why might the degree of negative valence and arousal interact? Valence and arousal have been shown to interact in determining behavior. For example, responses to negative words or pictures are faster if they are high in arousal [41–43]. This is because when combined with strong negative valence, high arousal indicates a particularly dangerous stimulus [44], which is best avoided and so reacted to faster. In marketing, an amplified avoidance response has also been demonstrated when a negative stimulus is combined with high arousal. This negatively influences measures such as the time spent in-store [45,46] and service satisfaction [37]. For example, when Mattila and Wirtz [37] increased arousal in an unpleasant service environment, it led to greater avoidance and thus worse service satisfaction ratings, whereas in a regular service environment, increased arousal led to better service satisfaction ratings.

Whether arousal and negative valence align positively may therefore depend on the extremity of negative valence. If the brand name is extremely negative and arousing then it should trigger a clear avoidance response because of the association with danger and threat and lead to poor evaluations. In contrast a moderately negative brand name should produce a milder avoidance response. This might not be enough to produce large negative effects on evaluations. Moreover, a slight avoidance response when combined with high arousal might make the brand name appealing. Arousal signals excitement and the slight feeling of danger created by the negative valence might heighten this feeling of excitement. Levels of arousal and valence may therefore have interactive effects on product evaluations.

## Study 2

Study 2 examined how the degree of negative valence and arousal of a brand name influences brand name evaluation. As reviewed above, we expected that extremely negative brand names may trigger an avoidance response and so lead to worse evaluation, especially when the brand name is also arousing. In contrast, the milder avoidance response created by moderately negative brand names might be beneficial when combined with arousal, as a slight impression of danger might add to the feeling of excitement created by the arousal. To test this idea, a set of brand names were selected that varied in valence and arousal, in a 3 (valence: extremely negative, moderately negative, non-negative) × 2 (arousal: high, average) within-participants design. We used a number of brand names in each category to ensure generality of the effect. Brand names from each category were then paired with a set of products with neutral valence, and participants indicated how much they liked the branded product and how willing they would be to buy it.

### Method

#### Participants

Participants were 42 students at a typical European university who participated voluntarily as part of a class exercise. All participants were over 18 and the majority under the age of 25, studying in English for a postgraduate degree. The experiment materials and instructions were all in English.

#### Materials and Stimuli

Brand names were sampled from ANEW (Bradley & Lang, 1999), The selected brand names were paired with 5 non-negative low arousal words denoting common objects, which served as the product category. The mean valence (V) and arousal (A) of the brand names in each category was as follows; non-negative valence average arousal (V = 4.96, A = 4.47), non-negative valence high arousal (V = 4.70, A = 6.33), moderate negative valence average arousal (V = 3.03, A = 4.41), moderate negative valence high arousal (V = 3.04, A = 6.59), high negative valence average arousal (V = 1.74, A = 4.52), high negative valence high arousal (V = 1.81, A = 6.56). For each of the categories with negative brand names there were 5 brand-product pairings. For each of the categories with non-negative valence brand names there were 10 brand-product pairings (see Table 3). Thus there were equivalent numbers of non-negative and negative brand names.

**Table 3:** Brand names and products in study 2.

Arousal	Valence	Brand Name	Product	Valence	Arousal	Log Frequency
High	Extreme Negative	torture	kettle	1.56	6.1	8.92
		slave	clock	1.84	6.21	9.48
		tragedy	lamp	1.78	6.24	8.2
		hatred	hat	1.98	6.66	8.78
		nightmare	bowl	1.91	7.59	9.04
	Moderate Negative	venom	kettle	2.68	6.08	8.43
		surgery	clock	2.86	6.35	9.02
		snake	lamp	3.31	6.82	8.56
		hurricane	hat	3.34	6.83	8.49
		trouble	bowl	3.03	6.85	10.81
	Non-Negative	volcano	kettle	4.84	6.33	8.02
		cliff	clock	4.67	6.25	8.54
		lightning	lamp	4.57	6.61	9.48
		startled	hat	4.5	6.93	7.12
		shotgun	bowl	4.37	6.27	8.15
		defiant	kettle	4.26	6.1	7.02
		rifle	clock	4.02	6.35	9.02
		doctor	lamp	5.2	5.86	10.37
		lion	hat	5.57	6.2	8.95
		noisy	bowl	5.02	6.38	7.92
Average	Extreme Negative	gloom	kettle	1.88	3.83	8.05
		depression	clock	1.85	4.54	8.95
		death	lamp	1.61	4.59	11.26
		grief	hat	1.69	4.78	8.27
		poverty	bowl	1.67	4.87	8.93
	Moderate Negative	fault	kettle	3.43	4.07	9.9
		waste	clock	2.93	4.14	10.21
		ignorance	lamp	3.07	4.39	9.43
		allergy	hat	3.07	4.64	7.92
		neglect	bowl	2.63	4.83	7.56
	Non-Negative	journal	kettle	5.14	4.05	10.28
		scissors	clock	5.05	4.47	7.22
		hammer	lamp	4.88	4.58	8.81
		cannon	hat	4.9	4.71	8.93
		swamp	bowl	5.14	4.86	8.65
		stove	kettle	4.98	4.51	7.52
		trunk	clock	5.09	4.18	8.09
		glass	lamp	4.75	4.27	9.94
		razor	hat	4.81	5.36	8.09
		curtains	bowl	4.83	3.67	6.8

To confirm the manipulations of valence and arousal, a 3 (valence; non-negative, moderately negative and extremely negative) x 2 (arousal; average and high) between subjects ANOVA was conducted on valence ratings and arousal ratings. For valence, there was a significant effect of valence only, *F*(2, 34) = 383.76, *MSE* = .087, *η*^*2*^ = .958, *p* < .001. For arousal ratings there was a significant main effect of arousal only *F*(1, 34) = 216.49, *MSE* = .170, *η*^*2*^ = .664, *p* < .001. The same analysis on word length and log word frequency yielded no significant main effects or interactions. Thus, different groups of brand names differed either in arousal or valence, but were equal in length and log frequency

#### Design and Procedure

Stimuli were presented in PowerPoint, with the brand in red and italics and the product in black and normal font, ensuring that the brand name and the product were clearly distinguishable. Participants were instructed which was the brand and which was the product, and were told that they would have to make several judgements about the branded product. Each brand-product pair was shown for 13 seconds, allowing time for consideration and production of responses. When each brand-product pair was presented, respondents indicated how much they liked the branded product and how likely they would be to buy it on a scale of 1 (not at all) to 7 (extremely). The 40 branded products were presented in random order.

## Results and Discussion

**Fig 2:**
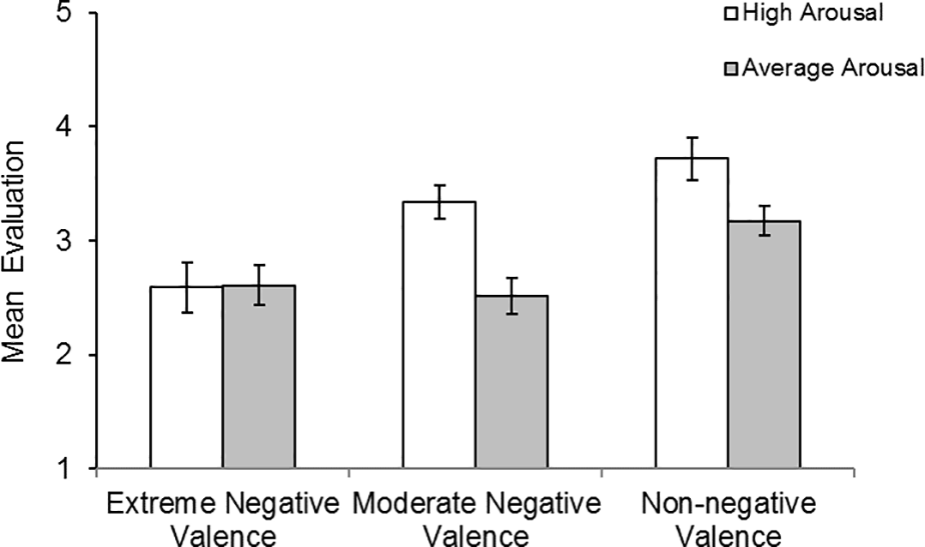
Mean evaluation ratings for each category of brand names in study 2. Potential evaluation scores range from 1 (low) to 9 (high).

Liking ratings and willingness to buy ratings were highly correlated (r = .70, *p* < .001) and thus were combined into an overall evaluation rating. Mean evaluation ratings for each category of brand name are shown in Fig 2. There was a clear effect of brand name valence, with worse evaluations for products with negative brand names. Moreover, evaluations worsened as brand name valence became more negative. For brand names with high arousal, there was a gradual decline in evaluations as brand names became more negative. In contrast, when arousal was average, there was a large initial decrease in evaluation as valence changed from non-negative to moderately negative, but no subsequent change in evaluation as valence became more negative. Notably, evaluations were equally poor for all classes of negative brand name except those that were moderately negative and highly arousing.

A 3 (valence) × 2 (arousal) repeated measures ANOVA indicated significant main effects of valence, *F*(2, 82) = 23.84, *MSE* = .82, *η*^*2*^ = .368, *p* < .001, and arousal, *F*(1, 41) = 20.94, *MSE* = .61, *η*^*2*^ = .338, *p* < .001, as well as a significant interaction, *F*(2, 82) = 10.05, *MSE* = 3.89, *η*^*2*^ = .197, *p* < .001. Simple main effects analysis (with Bonferroni adjustments) showed that there was a significant influence of arousal on non-negative brand names (*p* < .001) and moderately negative brand names (*p* < .001), but not extremely negative brand names (*p* = .901). For high arousal brand names, evaluations significantly differed between all types of negative brand name (all *p*<036). For average arousal brand names, evaluations were significantly better for non-negative valence brand names compared to moderately negative (*p* < .001) and extremely negative (*p* < .001) brand names. Moderately negative and extremely negative brand names did not differ in their evaluations (*p* = 1.00),

Two interpretations of the interaction between valence and arousal are possible. The first is that something about the extremity of valence renders the manipulation of arousal ineffective. For example, extremely negative brand names could trigger an avoidance response large enough that it cannot be modulated by arousal. Although plausible, this explanation of the interaction does not explain why moderately negative and highly negative brand names were evaluated similarly when arousal was low. Closer inspection of the data indicates that the effect of negative valence on ratings was similar for all classes of negative brand name except one, brand names that were highly arousing and moderately negative (e.g., Venom). Thus an alternative explanation for the interaction is that there is a general negative effect of negative valence but that something about the combination of moderately negative valence with high arousal renders these brand names relatively immune to this. Indeed, not only were moderately negative high arousal brand names evaluated much better than the other classes of negative brand names, but they seemed to be evaluated at similar levels to brand names with non-negative valence and average arousal (e.g., Hammer). Thus although evaluations for moderately negative high arousal brand names were significantly lower than non-negative high arousal brand names (as indicated above) they did not significantly differ from non-negative average arousal brand names (*t*(41) = 1.32, *p =* .38). So whether a negative brand name negatively impacts on evaluation depends on its extremity and its level of arousal.

Overall study 2 demonstrates that, except for moderately negative high arousal brand names, all other classes of negative brand name have a negative effect on evaluation. However, it is possible that such effects occur only at initial exposure to a negative brand name and decline with additional exposures. As such, one limitation of study 2 is that it does not examine changes in evaluation over repeated exposures. For example, whilst the brand name *Fat Fac*e (a clothing store in the UK) may initially provoke negative feelings, repeated exposure to the brand name may reduce these negative feelings or reduce the likelihood that any such feelings elicited carry over to evaluations of the branded product. Such *affective habituation* [47,48] is distinguishable from the mere exposure effect which is when repeated exposure to new stimuli increases liking of these stimuli [49,50] a finding also shown with repeated exposure to advertisements and brand liking [6,51]. Evaluations of a branded product should therefore improve with repeated exposure to a brand name regardless of the type of brand name. Thus Kohil et al [6] show that liking of a branded product improves with repeated exposures regardless of whether a brand name is meaningful (where the brand name is informative such as CleanAll kitchen cleaner) or non-meaningful (where the brand name is not informative such as Alcon kitchen cleaner). In contrast, affective habituation should only influence evaluations for brands that are negative in valence, and should have stronger effects the more negative the brand name [47]. In study 3 we therefore examine how repeated exposure to brand names over the course of two weeks influences evaluation of branded products. In addition, unlike study 2, different sets of products were used in each subcategory of brand names. This method was used in order to increase generality of the findings of study 2 and show that these effects did not simply arise due to the set of products used.

## Study 3

### Method

#### Participants

Participants were 48 students at a typical European university who participated voluntarily as part of a class exercise. All participants were over 18 and the majority under the age of 25, studying in English for a postgraduate degree. The experiment materials and instructions were all in English.

#### Materials and Stimuli

Brand names used in study 2 were randomly paired with neutral valence, low arousal words denoting common objects taken from the ANEW, and these latter words served as the products. This approach differed to that of study 2 in which the same products were used in all brand name classes. This method was used in order to examine the generality of the effects in study 2 by showing that the findings could be replicated with different brand name and product assignments. Moreover, using different products in each category would show (if data are generally consistent with that of study 2) that the evaluations were largely determined by the brand name. In total there were 5 brand-product pairings in each of the brand name categories (see Table 4). The category of non-negative brand names with high arousal was not used in this study, primarily because the focus was on negative brand names. That is, because the study focused on affective habituation for negative stimuli it was considered necessary only to contain a single class of non-negative brand name.

**Table 4:** Brand names and products in study 3.

	Extreme Negative		Moderate Negative		Non-negative Valence	
Arousal	Brand	Product	Brand	Product	Brand	Product
**High**	torture	glass	venom	clock		
	slave	butter	surgery	kettle		
	tragedy	chair	snake	tool		
	hatred	violin	hurricane	trunk		
	nightmare	razor	trouble	hat		
**Average**	gloom	bowl	fault	truck	journal	appliance
	depression	curtains	waste	cord	scissors	vest
	death	basket	ignorance	machine	hammer	fork
	grief	stove	allergy	table	cannon	lamp
	poverty	jug	neglect	cabinet	swamp	pencil

#### Design and Procedure

Evaluation ratings for each brand name and product pairing were made using the same method as study 2. Respondents provided ratings at 3 different time points, once in the morning (T1), once the same afternoon (T2), and once two weeks later (T3).

### Results and Discussion

**Fig 3:**
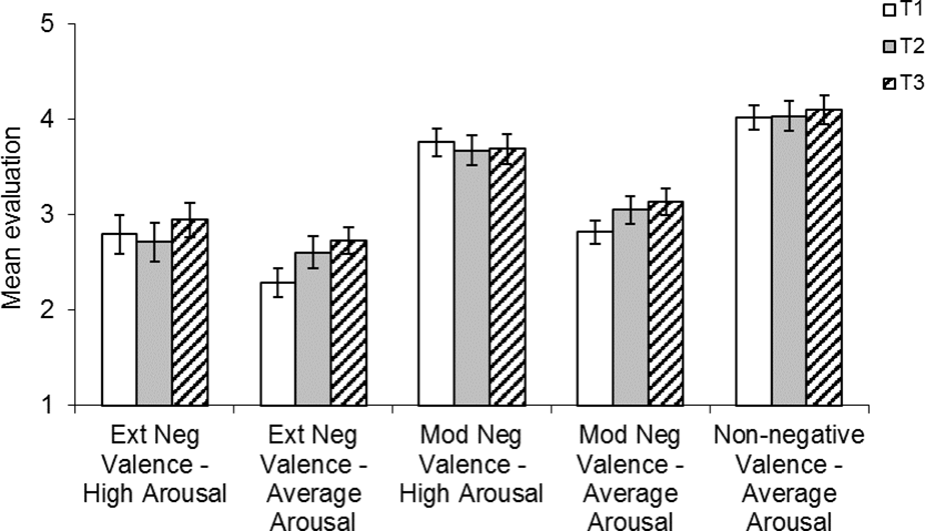
Mean evaluation ratings for each category of brand name at t1, t2 and t3 in study 3. Potential evaluation scores range from 1 (low) to 9 (high).

Liking ratings and buying intention ratings were highly correlated (r = .74, *p* < .001) and so, as in study 2, they were averaged to form a single evaluation rating. Mean evaluation ratings for each category of brand name at each time point are shown in Fig 3. The results were strikingly similar to study 2, despite products differing between the different sub categories of brand names.

A 5 (condition; extreme valence average arousal, extreme valence high arousal, moderate valence average arousal, moderate valence high arousal and non-negative valence average arousal) x 3 (time; T1, T2 or T3) repeated measures ANOVA on evaluation ratings indicated a significant main effect of condition, *F*(4, 184) = 43.38, *MSE* = 2.17, *η*^*2*^ = .495, *p* < .001, and a significant interaction between condition and time, *F*(8, 368) = 2.30, *MSE* = .56, *η*^*2*^ = .05, *p* = .038 (Greenhouse Geisser corrections reported). Simple effects analysis (all simple effects analyses used a Bonferroni correction) between the five conditions revealed a number of similarities with study 2. Negative valence had a detrimental effect on evaluation, with the non-negative average arousal brand name group receiving significantly higher evaluations than all other groups (all *p* < .006). Again, the moderately negative high arousal brand names were evaluated significantly better than all other classes of negative brand name (all *p* < .006). In addition, no difference was observed between evaluations for the two extreme negative valence conditions (*p* = .213). Interestingly in study 3, extremely negative average arousal brand names also received worse evaluations than moderately negative average arousal brand names (*p* = .002). This was not the case in study 2 and indicates that the degree of negative valence is important.

To investigate the interaction between condition and time, simple effects analysis compared T1 and T3 evaluations across each condition. This revealed that increasing the number of exposures had a positive effect on evaluations for extremely negative brand names with average arousal (*p* = .010) and moderately negative brand names with average arousal (*p* = .017). For all other groups of brand name there were no significant differences between evaluations between any of the time points. Thus only negative brand names with average arousal benefitted from multiple exposures. Polynomial contrasts across T1, T2 and T3 were completed for both these groups and revealed a significant linear improvement in evaluations for brand names with extreme negative valence and average arousal, *F*(1, 47) = 9.05, *MSE* = .51, *η*^*2*^ = .162, *p* = .004, and a significant linear improvement in evaluations for brand names with moderate negative valence and average arousal, *F*(1, 47) = 7.57, *MSE* = .32, *η*^*2*^ = .139, *p* = .008. The effect of time was therefore evident for some classes of brand name only. Importantly, if the effect were simply attributable to mere exposure, then evaluations should have improved across exposures in all conditions and in the non-negative condition. Thus it appears that affective habituation does occur for negative brand names, but only when they are of average arousal.

To summarize, the majority of the effects of manipulating brand name valence and arousal observed in study 2 were replicated. This is despite the fact that the products differed between the subcategories of brand name. In both study 2 and 3 multiple brand names were used in each brand name subcategory to prevent any systematic effect of the relation between the brand name and the product on evaluation. In study 2 the same products were used in each subcategory of brand name so that differences in evaluations could not be explained by differences between products. However this raises the question of whether the effects observed were due to the limited set of products used. In study 3 a completely different set of products were used that varied between subcategories of brand name. The replication of the main findings of study 2 therefore indicates that the effects of study 2 were not due to the set of products used, nor were they due to particular relations between brand name and product.

Combined, the results of studies 2 and 3 provide strong evidence that the valence and arousal of a brand name can have systematic effects on evaluations of branded products. In addition, study 3 shows clear evidence that evaluations improve as the number of exposures to the brand name increase. Importantly, this improvement in evaluation was not observed for all types of brand names, suggesting that the improvement was not due to the mere exposure effect [6]. Rather, the improvement observed was limited to average arousal brand names for which the negative valence had a large impact on evaluation, which is more consistent with affective habituation.

## General Discussion

New brands as well as brand extensions are continually entering the market using words with negative valence as brand names in many different product categories (e.g., *Fat Bastard* wine, *Burn* energy drink, *Criminal* clothing and *Urban Decay* cosmetics). Given that the usage of negative brand names is not a new phenomenon (e.g., Dior’s *Poison* perfume) and the effects of brand names on product perceptions and evaluations are well-established [6,8,52,53], it is surprising that the effects of brand name valence (and how to influence them) have not been studied. Certainly name valence is known to be important, with the valence of a person’s name influencing behaviours such as whether someone is befriended on facebook [54] and the positive valence associated with one’s own name producing preference for brands that contain name letters [55]. In three studies we therefore examined what effects using a negative brand name might have on memory and evaluation.

Study 1 demonstrates that when putting negative and non-negative brand names in competition by presenting them in the same display, negative brand names are better recognised relative to non-negative brand names, as are surface characteristics of the brand name, in this case the colour font. This extends our understanding of brand names in marketing in important ways. First, we use real, existing examples of negative brand names. By showing that brand name valence has similar effects on memory as that reported for word valence in cognitive psychology [23] we generalise these previous findings. Second, brand names are not normally presented in isolation, but are typically presented alongside logos and other visual information. Presenting brand names alongside logos, we demonstrate that recognition is superior *also* for these logos, indicating that that the effect of word valence on memory is not simply due to the valence influencing processing of the word, but extends to other visual information surrounding the word [26]. As such, negative brand names can be instrumental when attempting to draw attention to, and increase memory of, adjacent visual information such as logos, and should therefore be taken into account when determining the placement of a negative brand name on advertising or on packaging.

Although both the effects on logo recognition and colour recognition were shown to be independent of arousal, the effect of valence on word recognition itself was not. It is important to note that data analysis here was completed on a more restricted set of data (e.g., item analysis rather than participant analysis) which may have led to these inconclusive findings for word recognition, as no clear effects of arousal were found either. We do not see this issue as particularly problematic for several reasons. First, valence effects on recognition memory have been shown to be independent of arousal in the literature [20,21]. These studies have used a much larger stimulus set (e.g., 2507 words [21]) than that used here and so not conclusively finding this effect in our more limited word recognition data does not mean that this effect does not exist. Second, we did find evidence of independent effects of negative valence on logo memory and font memory supporting the general hypotheses that negatively valenced stimuli do capture attention and improve memory. Of course, it is possible that the negative words themselves capture attention but this attention was then diverted to the logos. Speaking against this interpretation is the evidence for an independent effect of valence for word colour memory, which indicates that attention was given to the word itself. In any case, study 1 was intended as a replication of a standard effect but using negative brand names and using logos. At the very least, it shows that negative brand names improve memory for associated logos, which is an important extension of this line of research.

Studies 2 and 3 examined the impact of negative valence on evaluation of a branded product. Moreover, they further explored the negative-brand-name phenomenon in two ways. First, by examining how the degree of negative valence influences evaluation and second, by disentangling the effects of valence from that of arousal, both of which determine affect [30,56,57]. The studies used different pairings of brand names and products yet yielded highly similar findings. Perhaps unsurprisingly, increasing negative valence of a brand name led to worse evaluations of a branded product. We suggest that the negative impact of negative brand names arises due to an avoidance response generated from the automatic appraisal of negative information as threatening and the automatic activation of negative information associated with the negative word [4,5,27].

Importantly studies 2 and 3 pointed to a key boundary condition: arousal. In both studies, moderately negative brand names were evaluated at similar levels to non-negative brand names when they were high in arousal. In comparison, the other classes of negative brand name received relatively poor evaluations. Clearly then, there are a subclass of negative brand names for which evaluations are surprisingly good. The key conditions appear to be that the negative valence is mild enough such that a strong avoidance response is not induced and that it is combined with arousal. The arousal should produce excitement and the negative valence may add to this by making the branded product seem slightly daring. Without high arousal, a negative brand name is simply unexciting and the avoidance response and any negative associations activated by the brand name appear to dominate evaluation.

Overall then, we have shown that there are several clear effects of using negative brand names. They may be more memorable themselves and also make visual information presented with them more memorable. However, most classes of negative brand name have negative effects on evaluation, the exception being when they are moderately negative and highly arousing. Although it is possible that with repeated exposure, people become habituated to the negative affect, this was only evident when brand names were of average arousal. It is possible that affective habituation would be demonstrated for the other classes of brand name, but that such affective habituation takes more exposure or more time as we only examined the effects of three exposures across a two week period. Nevertheless, at the very least it is important for marketers to be aware that affective habituation occurs more quickly for negative brand names with average arousal. Importantly, even with such affective habituation, the improvements in ratings were modest by the third presentation in a week, such that these classes of brand name still received much lower evaluations.

What are the implications of these findings? During brand name selection the valence and arousal of the proposed brand name relative to other products in the consideration set (i.e., the brand names of rival products that you would consider purchasing instead of the proposed product) should be considered. If the consideration set comprises brand names that are non-negative and of average arousal, then using a moderately negative brand name with high arousal will not be detrimental. thus providing marketers with a robust lever to potentially increase brand awareness (memory of the brand name or brand logo) without detrimental effects on evaluation. As ever, some degree of caution should be taken in generalising these results given that the stimuli were presented relatively abstractly (brand name and product name) with no other information (e.g., an actual product, or a longer product description of the type provided in adverts). Nevertheless, as the first study into this phenomenon we believe the results constitute a good initial analysis that further work could progress. In addition, the current study focused on the comparison between negative and non-negative brand names. A comparison with positive brand names would be an important next step in providing a larger understanding of the effects of brand name valence. Like negative brand names, positive brand names also differ in arousal, and thus an assessment of the contributions of positive valence and arousal would also provide a much clearer understanding of the effects of brand name arousal on evaluations of branded products.

Despite negative valence having a detrimental effect on evaluations of branded products, an initial glance at the phenomenon of negative brand names does not necessarily reveal a detrimental effect of these brand names. For example, the brand name *fcuk* has been hugely popular. Undoubtedly some products with negative brand names are marketed at young adults. Nevertheless, the sample used here was university students, who are precisely the target audience of some of the negative brands, particularly with regard to clothing and fashion accessories, yet clear effects of negative valence were demonstrated. Indeed, it is possible that the effects demonstrated here may be amplified even further using another sample population, such as older adults. That said, there is a preponderance of negative brand names in the wine industry (e.g., *Sassy Bitch* or *Fat Bastard*) and luxury perfume industry (e.g., *Poison* or *Addict* by Dior, *Obsession* by Calvin Kline, *Egoiste* by Chanel), which are associated with a much broader (and wealthier) demographic, suggesting the appeal of negative brands may actually be a more widespread phenomenon.

It is also important to consider that negative brand names are probably having multiple effects at various different levels. Here we have attempted to examine the effects of brand name valence in isolation from more complex issues such as the relationship between brand and product. Thus, in studies 2 and 3 brands were randomly paired with products in order to rule out any influence of an association between the brand name and the product. This method enabled a direct assessment of the effect on evaluation and memory of negative valence itself. However, one of the potential functions of a brand name is to highlight positive, situation and context specific attributes of the product. For example, the battery brands *Eveready* and *Energizer* indicate that their batteries will be long lasting. Such meaningful brand names tend to be liked more than meaningless brand names [6,58]. Clearly some negative brand names are meaningful in the sense of highlighting positive attributes; *Burn* energy drink implies that when you drink this you will be ready to burn lots of energy and *Poison* perfume suggests that the perfume will have an intoxicating effect on those around you.

Other instances of negative brand names appear to highlight negative attributes, however, sometimes even using humour based appeals; thus *Fat Bastard* wine or the *Sweat Shop* cycle store could be considered a benign violation [59]. Yet, other negative brand names do not seem to share this attribute, for example *Monster* energy drink or Augusta’s *Brutale* motorbike. Negative brand names might also be associated with counter-norm behaviour, such as sin. Good examples of this are the *Poison* perfume or *Urban Decay* cosmetics, but counter examples exist such as *Big Ass* fans. Similarly many negative brands seem to be used for hedonic products, such as *Killer* clothing or YSL’s *Opium* perfume, but there are examples of less hedonic products, such as *Demon* internet provider or *Earthquake* outdoor tools. Consideration of these examples demonstrates that assessing the interplay between meaning and negative brand names, which is one component of negative brand use, is not straightforward. As the first inroad into this phenomenon we firmly believe that the most useful approach is to investigate, first and foremost, whether there are *general* effects of negative brand names on their appeal that arise due to the processing of their valence. Indeed, such an aim is consistent with prior research about brand names [8,60,61]. Throughout the studies, we therefore used a variety of brand names in each valence category and randomly paired them with products, in order that any impact of meaningfulness be reduced and a pure measure of the impact of brand name valence and arousal be assessed. Further research should however tease out potential effects of these additional variables.

## Supporting Information

S1 File Experiment 1 Raw Data. (XLSX)

S2 File Experiment 2 Raw Data. (XLSX)

S3 File Experiment 3 Raw Data. (XLSX)
